# Informing Potential Participants about Research: Observational Study with an Embedded Randomized Controlled Trial

**DOI:** 10.1371/journal.pone.0076435

**Published:** 2013-10-03

**Authors:** Helen M. Kirkby, Melanie Calvert, Richard J. McManus, Heather Draper

**Affiliations:** 1 Medical Research Council Midland Hub for Trials Methodology Research, University of Birmingham, Birmingham, United Kingdom; 2 National Institute for Health Research School for Primary Care Research, Primary Care Clinical Sciences, School of Health and Population Sciences, University of Birmingham, Birmingham, United Kingdom; 3 National Institute for Health Research School for Primary Care Research, Department of Primary Care Health Sciences, University of Oxford, Oxford, United Kingdom; 4 Medicine, Ethics, Society and History, School of Health and Population Science, University of Birmingham, Birmingham, United Kingdom; 5 Cancer Research UK Clinical Trials Unit, School of Cancer Science, University of Birmingham, Birmingham, United Kingdom; University Paris Descartes, France

## Abstract

**Objectives:**

To assess: 1) the feasibility of electronic information provision; 2) gather evidence on the topics and level of detail of information potential research participant’s accessed; 3) to assess satisfaction and understanding.

**Design:**

Observational study with an embedded randomised controlled trial.

**Setting:**

Low risk intervention study based in primary care.

**Participants:**

White British & Irish, South Asian and African-Caribbean subjects aged between 40-74 years eligible for a blood pressure monitoring study.

**Interventions:**

PDF copy of the standard paper participant information sheet (PDF-PIS) and an electronic Interactive Information Sheet (IIS) where participants could choose both the type and level of detail accessed.

**Main Outcome Measures:**

1) Proportion of participants providing an email address and accessing electronic information 2) Willingness to participate in a recruitment clinic. 3) Type and depth of information accessed on the IIS. 4) Participant satisfaction and understanding.

**Results:**

1160 participants were eligible for the study. Of these, 276 (24%) provided an active email address, of whom 84 did not respond to the email. 106 responded to the email but chose not to access any electronic information and were therefore ineligible for randomisation. 42 were randomised to receive the PDF-PIS and 44 to receive the IIS (with consent rates of 48% and 36%, respectively; odds ratio 0.6, 95% confidence interval 0.25 to 1.4). Electronic observation of information accessed by potential participants showed 41% chose to access no information and only 9% accessed the detail presented on the Research Ethics Committee approved participant information sheet before booking to attend a recruitment clinic for the intervention study. 63 of the 106 participants (59%) who chose not to access any electronic information also booked an appointment.

**Conclusions:**

Current written information about research may not be read, emphasising the importance of the consent interview and consideration of new ways of presenting complex information.

## Introduction

Potential participants of medical research need to be adequately informed about the risks, benefits and processes involved in order to make an informed decision regarding participation [1-6]. Normally this information is provided in the participant information sheet (PIS) and verbally reinforced in the consent interview [6]. Information provision in research is tightly controlled by regulatory codes [6-8] and the content of the PIS is reviewed by research ethics committees (RECs)[1]. Even with extensive guidance provided by the UK National Research Ethics Service (NRES) [1], UK researchers often find it difficult to decide on the level of detail to provide in a PIS[2].

The PIS must be understood by research participants if it is to aid decision-making[2,9]. Participants are known to struggle with scientific concepts such as ‘randomisation’, ‘equipoise’, ‘risk’ and ‘probability’. [10-12] The higher the reading level and the more jargon or technical language used, the less comprehensible is the PIS, and presentation and length are key factors in participant understanding [3,13,14].

Information should be provided that meets the needs of individuals and, when asked using hypothetical scenarios, people generally say they want more information[15-20]. The prevailing sense that ‘people want more rather than less information’ and meeting regulatory requirements may result in PISs are that are very long: the recommended template alone runs to 3 pages [1]. This length is now perceived as a deterrent to participation [1,21,22].

One way forward is to allow participants to choose the level of information they require to inform their decision, but there is limited evidence on the feasibility of such an approach or agreement on whether this would result in them being sufficiently informed. Antoniou et al. [15] developed an electronic ‘unfolding’ information sheet for potential participants of an Internet survey investigating the development of twins during early childhood. The amount of information participants accessed was recorded electronically. Only 9-18% of participants accessed information at the level suggested by a standard PIS; reported and actual patterns of information accessed differed significantly[15]. This suggests that asking potential participants what information they used to make a participation decision may not yield reliable data.

Building on the work by Antoniou et al [15], the aim of this study was to assess the feasibility of electronic information provision and gather evidence on the topics and level of detail of information potential participant’s accessed when deciding whether to participate in a piece of low risk interventional research.

## Methods

### Ethics

The Birmingham and Black Country Research Ethics Committee (reference 09/H1202/114) and relevant NHS authorities reviewed the study as part of the regulatory process for the parent study. The trial was not registered since it did not include a health outcome. The full trial protocol can be accessed by contacting the corresponding author.

### Study Design

The information provision (IP) study had three components. The first addressed the feasibility of electronic information provision, comparing electronic information provision to a standard paper PIS. The second was an RCT (IIS RCT) that compared a PDF version of the same standard PIS (PDF-PIS) with an interactive electronic information sheet (IIS). The third was an observational study of participants randomised to the IIS arm of the IIS RCT and recorded the information accessed by each participant. Data were collected between 5^th^ February and 12^th^ December 2011.

The IP study was embedded in a parent study (Blood Pressure Monitoring in Different Ethnic Groups [BP-Eth]) [23]. Participants of the parent study completed a short survey about blood pressure monitoring and were then asked to indicate if they would be interested in taking part in a study comparing blood pressure measuring methods. The outcome of the IP study was booking into a recruitment clinic for the parent study. Everyone expressing an interest in continued participation in the parent study (whether included in IP study or not) attended a recruitment clinic, during which the study was explained by a research nurse and any questions answered prior to formally consenting (or declining) to participate [23].

### Participants

**Figure 1:**
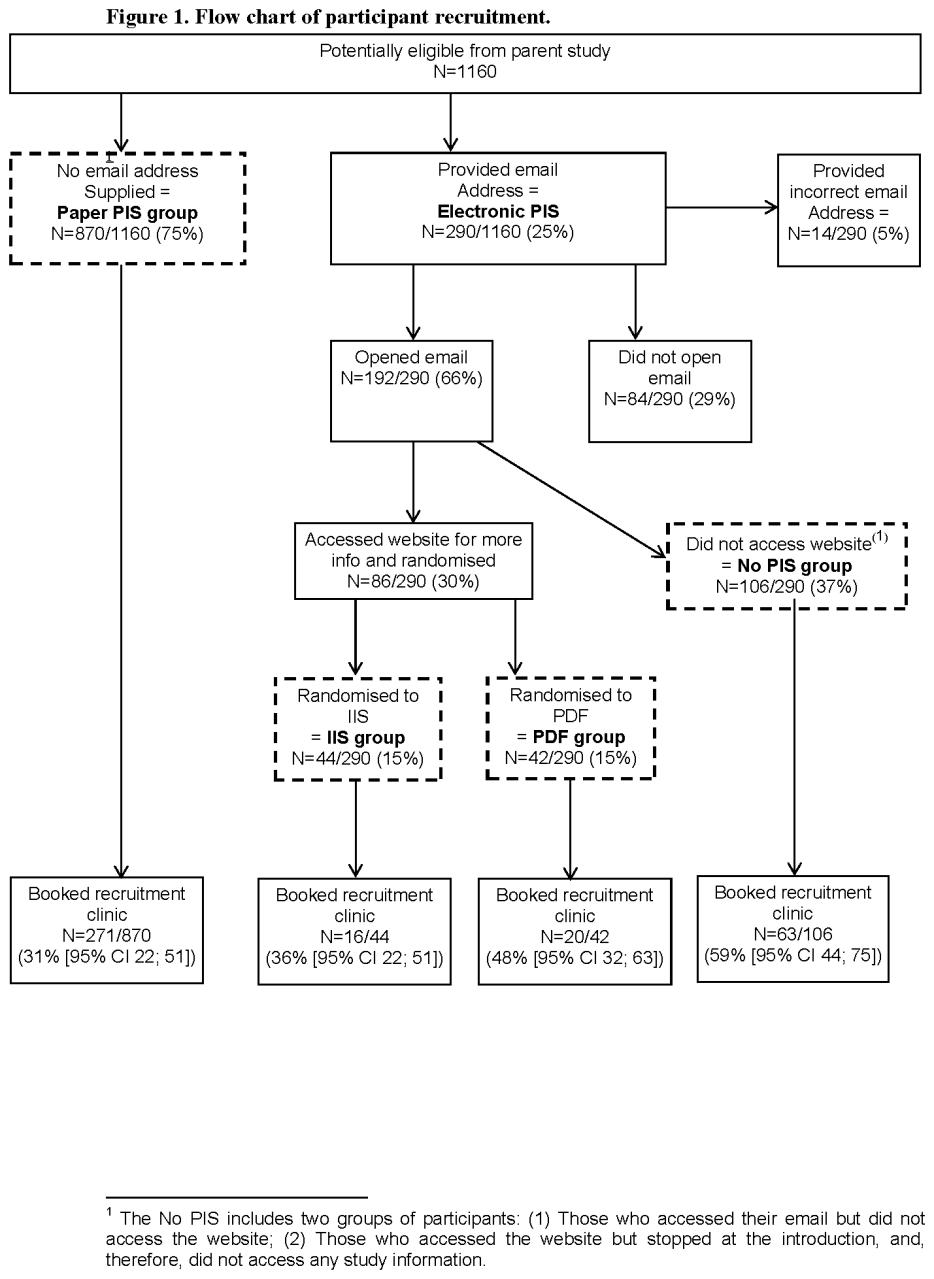
Flow chart of participant recruitment (prepared and submitted as an individual file).

Figure 1 shows how participants were recruited to each component of the IP study.

The feasibility component included all participants who wished to continue with the parent study. Demographic data were collected for the parent study, anonymised and made available to the IP study. Anyone who provided an email address was sent an electronic invitation with a link to the parent study website. If no email address was provided, a standard paper PIS was posted.

Participants were included in the parallel group RCT and randomised 1:1 to receive the PDF-PIS or IIS if they clicked the link to the parent study website, provided their age and gender (stratification variables) and indicated their willingness to participate in the IP study.

### Interventions

The PDF-PIS was an electronic copy of the parent study paper PIS that had been approved by an ethics committee (File S1).

The IIS was based on the unfolding PIS described by Antoniou et al. [15] and is available to view at http://mededweb1.bham.ac.uk/KirkbySite/. The IIS presented the reader with a screen containing a list of frequently asked questions (FAQ) about the parent study that corresponded to titles in the paper PIS. Behind each FAQ were up to three levels of increasingly detailed information. Compared with the paper PIS, level one contained less detailed information (minimal information), level two was an exact copy (standard information), and level three was more detailed (extended information). Level three also contained links to evidence in external resources. The level of study information accessed by each IIS randomised participant was recorded by an SQL database connected to the website.

### Sample size (of the IIS RCT)

Expert opinion of the effect size required to change practice was used to inform sample calculations [24]. With a predicted type I error of 5%, a baseline consent rate of 30% and a type II error of 10%, the calculated total sample size needed to determine an effect size (change in rate of participation in the next phase of the study) of 29%^20^ was n=132.

### Randomisation

The random allocation sequence was generated by HK and allocated by a central computer linked to the trial website. Block randomisation was stratified by age and gender. Willing participants enrolled themselves to the IIS RCT when they accessed the website. There was no blinding to the assignment of intervention.

### Outcomes

The primary outcomes for each phase of the study were as follows

1 Feasibility study: proportion of participants that provided an email address.
2 IIS RCT: booking to attend the recruitment clinic of the parent study was used as a proxy for being willing to consent.
3 Observational study: amount and type of information accessed.

Secondary outcomes included: level of understanding of, and satisfaction with, the information provided. Participant understanding of the information provided was assessed using the validated ‘Quality of Informed Consent tool’ (QuIC) [25] adapted for use in this study. The satisfaction questionnaire developed Antoniou et al. [15] was adapted to determine satisfaction with the information provided.

No changes were made to trial outcomes after trial commencement. The trial was stopped at ten months. This was due to funding constraints and because the parent study was moving to target ethnic minority groups where email addresses were not being regularly provided.

### Statistical methods

All analyses were carried out using SAS version 9.2.

### Feasibility study

Each participant was assigned an Index of Multiple Deprivation (IMD) score [26], based on postcode and calculated using the GeoConvert tool available through the official Census website [27]. Low IMD scores indicate high levels of deprivation.

The proportion of participants providing an email address, accessing electronic information and levels of understanding were estimated for each study group (IIS, PDF-PIS, no PIS) and exploratory analyses were undertaken based on patient demographics (age, gender, ethnicity, deprivation).

### IIS RCT

The IIS RCT analyses were based on the intention to treat principle. The primary analysis assessed the impact of the IIS on whether participants booked into recruitment clinics, using logistic regression accounting for age and gender. Results were presented as odds ratios with their corresponding 95% Confidence Limits (95% CL).

### Observational study

The potential participants’ use of the IIS was recorded to produce data about the information accessed by each individual. The proportion of participants accessing each level was calculated to show which types and levels of information were most/least often accessed.

The number of words the average person can read in one minute was used to determine how long it would take to read each piece of text on the IIS [28,29]. This calculation was used to estimate whether the participant had time to read the text or not. Adjusted analyses were undertaken to exclude data when the participant could not have read the full text based on the length of time it was accessed for.

### Understanding and satisfaction questionnaire

Everyone who booked an appointment for the recruitment clinic in the parent study and/or was included in the IIS RCT, was asked to complete a questionnaire to determine their understanding of the study and their satisfaction with the way in which information was provided.

## Results

Demographic data are shown in Table 1 and the number of participants included in each phase on the IP study is shown in Figure 1.

**Table 1:** Demographic Data.

	**Electronic or paper**		**Information provision study groups**		
	Email address	No email address (Paper PIS)	IIS*	PDF-PIS*	No PIS
**Age**					
Mean age (SD)	53·4 (9·3)	58·5 (9·5)	54·5 (9·3)	53·9 (9·8)	54·3 (9·7)
**Gender n(%)**					
Male	157/290 (54%)	430/870 (49%)	24/44 (55%)	25/42 (59%)	52/106 (49%)
Female	133/290 (46%)	440/870 (51%)	20/44 (46%)	17/42 (41%)	54/106 (51%)
**Ethnicity n (%)**					
White	172/290 (59%)	470/870 (54%)	31/44 (71%)	31/42 (74%)	57/106 (54%)
Asian	43/290 (15%)	175/870 (20%)	2/44 (5%)	4/42 (10%)	19/106 (18%)
Black	59/290 (20%)	197/870 (23%)	5/44 (11%)	5/42 (12%)	25/106 (24%)
**Deprivation**					
Mean IMD Score (SD)	35·4 (16·9)	38·6 (17·2)	34·6 (17·8)	34·3 (17·8)	38·2 (16·6)

* denotes a randomised group in the IIS RCT

### Feasibility Study

1160 parent study participants were eligible for the IP study. Of these, 276 (24%) provided an active email address, of whom 84 did not respond. 106 responded to the invitation email but did not access any electronic information. This resulted in an unanticipated study group (the no PIS group). 86 patients were randomised to receive electronic information.

Exploratory analyses suggested that those who provided an email address were on average younger, more commonly white and less deprived (Table 1). Also, those participants in the no PIS group were more deprived than those who accessed study information. The booking rates showed a trend towards increased booking in those contacted by email (odds ratio 1.25, 95% CL 1.0-1.7); the highest booking rate was observed in the no PIS group (Figure 1). Given the small sample size, these results should be treated with caution.

### IIS RCT

The rate at which participants booked a recruitment interview was reduced, although not significantly, by using IIS compared to PDF-PIS (16/44 [36·4%] and 20/42 [47·6%] respectively, OR=0·6 [95% CL 0·25; 1·4]) (Figure 1). These results should be interpreted with caution as the trial was underpowered to detect statistically significant differences.

### Observational study

41% (18/44) of those randomised to the IIS accessed no information and were added to the no-PIS group. Only 18% (8/44) participants accessed any information at the level provided in the paper PIS (level 2), of these 4 chose to access at least some additional information in level 3 for one or more FAQs (Table 2). The median time spent on accessing information was 57 seconds (IQR 0 to 195 seconds).

**Table 2:** levels of information accessed in the IIS arm of the RCT (N=44).

**Level of information**	**Accessing information under any FAQ n (%)**
No levels (had information but chose not to access any)	18 (41%)
Only needed level one	18 (41%)
Stopped at level two (level of information identical to UK REC approved paper PIS)	4 (9%)
Needed level three	4 (9%)
**Reading Time**	**Median time in seconds (IQR)**
Total time spent reading information	57 (0;195)

Participants were more likely to access information about the practical aspects of the study, such as the risks and benefits of taking part (Table 3).

**Table 3:** The proportion of participants who accessed each FAQ and stayed long enough to have read each level of information.

	Level one*		Level two*		Level three*	
Purpose of the study	17/44	39%	3/44	7%	1/44	2%
Have to take part	6/44	14%	0/44	0%	0/44	0%
Why been chosen	18/44	41%	4/44	9%	1/44	2%
Expenses	22/44	50%	5/44	11%	1/44	2%
What will happen	13/44	30%	0/44	0%	0/44	0%
Risks	22/44	50%	7/44	16%	2/44	5%
Benefits	20/44	46%	3/44	7%	0/44	0%
Problems	16/44	36%	1/44	2%	0/44	0%
Confidentiality	10/44	23%	1/44	2%	0/44	0%
Don’t want to carry on	6/44	14%	0/44	0%	0/44	0%
GP	14/44	32%	3/44	7%	1/44	2%
Samples	15/44	34%	n/a**		n/a**	
Genetic tests	10/44	23%	n/a**		n/a**	
Results	11/44	25%	1/44	2%	1/44	2%
Organising and funding	10/44	23%	3/44	7%	2/44	5%
Reviewed study	11/44	26%	2/44	5%	2/44	5%
Further info and contact	4/44	9%	n/a**		n/a**	
**Accessing any information type**	26/44	59%	8/44	18%	4/44	9%

* Participants who did not access level 1 could not access any of the later levels and participants who accessed levels 2 and 3 must have accessed the previous levels to do so and are therefore included in the figures for the previous level.

** n/a means there was no information available under that level

### Understanding and satisfaction

An understanding and satisfaction questionnaire was completed by participants in all groups: paper PIS n=165/271 (60.9%), PDF-PIS n=26/42 (61.9%), IIS n=41/44 (93.2%) and no PIS n=37/63 (58.7%). The level of understanding was similar regardless of how much information, if any, was accessed (Mean score out of 15: No PIS=11 [95% CI 10; 12]; IIS=11 [95% CI 10; 12]; PDF-PIS=11 [95% CI 10; 12]; Paper PIS=10 [95% CI 10; 11]). Participants across all study groups were generally satisfied with the level of information they received, regardless of how much they accessed.

Participants were less likely to want more information about the parent study if they received study information electronically; only 1/40 (2.5%) of those in the IIS and 2/24 (8.3%) in the PDF-PIS group wanted more information about the study compared with 28/137 (20.4%) in the paper PIS group. One person (1/36; 2.8%) who chose not to access the PIS before booking a recruitment clinic appointment stated that s/he would have liked more information about the study. IIS participants did not accurately recall what information they had accessed.

## Discussion

This study has demonstrated that in an ageing ethnically diverse group, electronic information provision may prove challenging as only 25% of potential research participants were willing to provide an email address and those that did sometimes provided inaccurate email address or did not respond.

Two striking sets of results emerged about the way in which information was used by potential participants at the point of invitation. First, a large proportion chose not to read any information about the parent study, and of these, 59% booked into a recruitment clinic, suggesting that they were willing in principle to participate in the parent study on the basis of the information provided in the invitation. Second, observed use of the IIS suggested that much of the information provided in the standard, REC reviewed paper information sheet was irrelevant in determining in principle willingness to participant: 82% of participants either accessed no information (41%) or only some of the information provided in level one (41%). Only 8/44 (18%) participants used information equivalent in level to that of the paper PIS (but not for every FAQ) and 4 (9%) participants wanted more information in at least one area. These findings are consistent with those observed by Antoniou et al. [15], where 88% of potential participants completed and submitted the web-based questionnaire without reading the level of information provided in a standard PIS, and between 28% and 30% chose not to access any information. This study and the work by Antoniou et al. are, as far as we know, the first to collect ‘real time’ data on the information read by potential participants.

Participant understanding was consistent across groups, including those who accessed no information. Participants who did not access information about confidentiality or ethical review still scored highly on the understanding questionnaire for these sections, suggesting that their answers were based on their general knowledge. If this assumption is correct, it may be sufficient for the standard PIS only to draw attention to information that is *contrary* to normal expectations; for example, when information will not to be kept confidential.

Participants were generally satisfied with the information they received. This suggests that not only did the majority of people decide about participation on the basis of very little information, but they did not want any more information than they read to make this decision.

Overall, our results suggest that for low risk studies, the current level of information provided contains more detail than participants want or are prepared to read. Catering for the majority, however, and providing less detailed study information risks providing insufficient information for a minority. Antoniou et al. [15] suggested allowing participants to choose what information to access, even if they choose to access less than is currently regarded as sufficient. They acknowledged, however, the danger that participants may thereby not access information that would have impacted on their decision-making. What constitutes sufficient information and who should decide this remains debatable. One promising avenue is the work by Blazeby et al. on core disclosure sets in routine clinical care, which could draw in a range of stakeholders (patients, clinicians, sponsors, regulators) in the context of research [30,31].

There are a number of ways in which information provision could be modified in other low risk studies. This would be most straightforward in e-trials where study information is already provided online and an IIS could be readily incorporated. It is, however, not always possible or desirable to provide information in an electronic format, and the outstanding issue remains that of determining what constitutes a sufficient level of information in the standard paper PIS. For higher risk studies, sponsors are likely to insist that all participants are provided with detailed information provision, whether or not they choose to read it.

Our study suggested that participants who accessed any information wanted at least some about the purpose of the study, risks and benefits; other information was rarely consulted despite conforming to regulatory codes (Table 3) [6-8]. The written information provided to potential participants in all low risk studies could, therefore, be radically streamlined to reflect these results, irrespective of whether it is provided in paper or electronic format. The opportunity to access more information either via a website or by requesting an additional lengthier paper PIS could be offered to those who want more information, and trained personnel would still need to be available to answer questions during consent interviews.

The majority of participants who were contacted electronically accessed no study information in the PIS before booking with a recruitment clinic, though it is possible that the information provided in the invitation letter was sufficient for them to reach this decision. If potential participants are not reading the information provided, attention should turn to the consent interview as the place to ensure that decisions are sufficiently informed. This may have implications for the process of ethical review, as it implies that the written information provided in the PIS is a less significant a safeguard to informed decision-making than the verbal information provided in the consent interview. Scrutiny of the intended verbal information may therefore be a more appropriate activity for review boards than scrutiny of the PIS. Certainly, consent received on the basis of sufficient verbal information would meet the researchers’ ethical obligations to the participant, particularly where this information was regularly revisited in a process of on-going consent. Whether it would provide researchers and sponsors with sufficient legal safeguards is debatable, as is the outstanding question of just how much information is sufficient reach an informed and enduring judgement about participation.

### Strengths and weaknesses of the Study

This prospective study provides data on the feasibility of contacting and informing potential research participants using electronic media and provides non-hypothetical data on the information accessed by those randomised to the IIS.

The age and characteristics of the participants may limit the generalisability of findings to other groups. Younger participants maybe more likely to utilise electronic information than the study population [32,33]. Further, the parent study may be regarded as a ‘low risk’ observational study and the findings are not, therefore, readily applicable to studies where the participants are more vulnerable due to their underlying condition or impaired mental capacity, or where the proposed interventions are accompanied by risks of major morbidity, mortality and reduced health-related quality of life.

Observed consistency in participant understanding across groups may reflect their general knowledge, but could also indicate that the questionnaire was insufficiently sensitive. Furthermore understanding and satisfaction were not assessed in those receiving the paper based PIS or no PIS if they did not book a consent appointment. These potential participants may have had less satisfaction or decreased understanding.

Participants in this study were not randomised to electronic or postal information provision and those with email addresses may be more likely to participate in research because of systematic differences to those without an email address.

### Suggestions for future research into information provision

These data suggest that further studies are required to determine how and what information is used by people who are invited to participate in clinical research, including that which is regarded as higher risk. Such studies need to reflect actual rather than intended or recalled behaviour, and should also include whether, how and why information provided at the time of consent is referred to later on if someone agrees to participate. This latter data would be useful in determining the extent to which detailed, extensive, written information is necessary from the perspective of the research participant.

## Conclusions

A significant proportion of our participants booked into the recruitment clinic without reading any information provided in the PIS in either format (PDF/IIS). This suggests that the recruitment interview remains vital to ensuring that participants have sufficient information before they are enrolled in studies. In our study, understanding was not influenced by the information provided, suggesting that at least some information regularly included in the standard PIS is unnecessary, unless it draws attention to a departure from usual practice. The majority of our participants accessed much less detailed information on a narrower range of topics than provided in the standard PIS. One inference is that information provided prior to the consent interview could be considerably streamlined in low risk studies. This leaves open the questions of whether the attention of ethics committees might be more appropriately focussed on scrutiny of intended verbal information, and what purpose is served by more detailed written information in such studies.

## Supporting Information

Checklist S1 **CONSORT Checklist.**
(DOC)

File S1 **An electronic PDF version of the parent study PIS approved by ethics.**
(PDF)
