# Supplementary material for: Informing Potential Participants about Research: Observational Study with an Embedded Randomized Controlled Trial
Source: PLoS One. 2013 Oct 3;8(10):e76435. doi: 10.1371/journal.pone.0076435 (PMC3789820; doi:10.1371/journal.pone.0076435)
Supplement: File S1 — An electronic PDF version of the parent study PIS approved by ethics. (PDF) [file pone.0076435.s002.pdf]

Patient information sheet

Blood pressure monitoring in different ethnic groups: Phase 2.

Part 1

You are being invited to take part in a research study about the different ways of measuring blood pressure. Before you decide if you are willing to get involved, it is important for you to understand why the research is being done and what it will involve. Please take time to read the following information carefully and discuss it with your friends and relatives if you wish. Ask us if there is anything that is not clear or if you would like more information.

What is the purpose of the study?

Blood pressure can be measured in a number of different ways. The most common method is to have it measured at your GP practice by your doctor or nurse. However, it is also possible to measure it yourself at home, or to wear a cuff for a day with a small machine which will measure it for you. Research has shown that blood pressures measured at home are likely to be lower than those measured at the GP practice. However, no studies have been done to look at whether the differences between blood pressure measured at home of at the GP surgery are the same for different ethnic groups (for example White British, Asian, White Irish, African-Caribbean): this is what we will be looking at in this study.

Why have I been chosen?

The study will involve 800 people from four different ethnic groups (White British, Asian, White Irish, African-Caribbean). You kindly expressed your interest in taking part when you returned our initial questionnaire, and we have chosen you on the basis of this.

Do I have to take part?

It is up to you to decide whether or not to take part. If you decide that you would like to get involved then we will ask you to sign a consent form. We will need to let your GP know that you are involved, and will also ask for your permission to access your medical records at your GP's practice. Once the study has started you are still free to withdraw at any time without giving a reason: this will not affect the care that you receive from your GP.

What will happen to me if I take part?

You will be involved in the study for a period of ten days. This will involve three visits to our research nurse / facilitator, as follows:

Visit 1(60 minutes) – You will initially come in to have your blood pressure measured by the nurse and to answer a questionnaire. You will then be taught to measure your own blood pressure at home: you will need to do this both in the morning and the evening for the next seven days and we will lend you equipment to do this. The equipment will store the readings that are taken, but you will also need to write them down.

Visit 2 (30 minutes) – You will return the home monitoring equipment and have your blood pressure taken by the nurse again. You will then be fitted with a cuff which will measure your blood pressure automatically over the next 24 hours: this should not interfere with your daily activities.

Visit 3 (30 minutes) - You will come back to return the cuff, have your blood pressure taken by the nurse for the last time and complete a final questionnaire.

Expenses and Payments
Any travelling expenses you incur will be refunded for this study.

What are the possible benefits of taking part?
The information that we get from this study will hopefully help to treat future patients with high blood pressure more effectively. In addition, your taking part will give us some really accurate information about your blood pressure: this will be passed on to your GP. Should the results suggest that you need treatment for high blood pressure (or a change in treatment if you are already taking it) then your GP will let you know.

What are the risks of taking part?
The study does not involve any long term risks to you. Some people may find having their blood pressure measured uncomfortable.

What if there is a problem?
Any complaint about the way you have been dealt with during the study or any possible harm you might suffer will be addressed. The detailed information on this is given in Part 2.

Will my taking part in this study be kept confidential?
Yes. We will follow ethical and legal practice and all information about you will be handled in confidence. The details are included in Part 2. If you agree to take part then you will be given a copy of this information sheet, and a signed consent form for you to keep.

If you require any further information or would like to discuss any issues that you may have then please contact:- Prof. Richard McManus 0800 234 6 432

Part 2

What will happen if I don't want to carry on with the study?
You are free to withdraw from the study at any time without affecting your treatment by your GP. If you withdraw from the study, we will need to use the data collected up to your withdrawal.

What if there is a problem?
If you have a concern about any aspect of this study, you should ask to speak to the researchers who will do their best to answer your questions: Prof. Richard McManus (**T: 0800 234 6 432**). If you remain unhappy and wish to complain formally, you can do this by contacting Ms Sarah Bathers, Primary Care Clinical Trials Unit Manager, Primary Care Clinical Sciences, University of Birmingham, Edgbaston, Birmingham B15 2TT (T: 0121 414 3323).

In the event that something does go wrong and you are harmed during the research and this is due to someone's negligence then you may have grounds for a legal action for compensation against The University of Birmingham but you may have to pay your legal costs.

Will my taking part in this study be kept confidential?
The study data will be collected by questionnaire, measurement (e.g. blood pressure, weight) and from your medical records (e.g. past medical history, current medications). Under the Data Protection Act of 1988, all personal data (names and addresses for example) will be stored securely at the University of Birmingham overseen by Prof. Richard McManus. Personal data (names and addresses) will be kept separately from study results. Anonymised data from the study will be used in the main analysis and may be used in future work, for example comparing blood pressure levels in different parts of the country.

Personal data (names and addresses) will be only accessible by authorised persons such as researchers, sponsors, regulatory authorities and for R&D audit (for monitoring of the quality of the research) and will be retained for approximately three years (as long as needed to inform participants of the results of the study). It will then be disposed of securely.

Involvement of your General Practitioner/Family doctor (GP)
We will ask your permission to inform your GP regarding the results from the study. If you do not give permission for this then it will not be possible to include you in the study because we want to be sure that you have appropriate follow up from your GP should for example your blood pressure be found to be high.

What will happen to any samples I give?
This study does **not** involve the taking of blood or other samples

Will any genetic tests be done?
No

Who is organising and funding the research?
The research is organised by the University of Birmingham. It is funded by the Research for Patient Benefit Programme which is part of the National Institute for Health Research and is funded by the Department of Health.

What will happen to the results of the research study?
We are hoping to publish the results of this study in one or more medical journals and to present them at one or more conferences. We will also write to you with the main findings of the study after it has finished unless you do not want us to. We will not identify you in any report/publication unless you have specifically given your consent for this.

Who has reviewed the study?
All research in the NHS is looked at by independent group of people, called a Research Ethics Committee, to protect your interests. This study has been reviewed and given favourable opinion by The Black Country Research Ethics Committee.

Further information and contact details
Further information is available as follows:

1. General information about research is available from the National Institute of Health Research Website: **http://www.nihr.ac.uk/**

2. Specific information about this research project can be obtained from Professor Richard McManus or one of the study team on the study helpline: **0800 234 6 432**

3. Advice as to whether you should participate can be gained with from your own GP or from Prof. McManus on the number above.

4. If you are unhappy with the study then please contact: Ms Sarah Bathers, PCCRTU Manager, Primary Care Clinical Sciences, University of Birmingham, Edgbaston Birmingham B15 2TT. 0121 414 3323.
